# The effect of floods on anemia among reproductive age women in Afghanistan

**DOI:** 10.1371/journal.pone.0191726

**Published:** 2018-02-09

**Authors:** Hamid Reza Oskorouchi, Peng Nie, Alfonso Sousa-Poza

**Affiliations:** 1 Institute for Health Care & Public Management, University of Hohenheim, Fruwirthstr. 48, Kavalierhaus 4, 70599 Stuttgart, Germany; 2 Department of Economics and Management, University of Florence, Florence, Italy; 3 Department of Economics and Management, University of Trento, Trento, Italy; 4 IZA, Bonn, Germany; University of Miami, UNITED STATES

## Abstract

This study uses biomarker information from the 2013 National Nutrition Survey Afghanistan and satellite precipitation driven modeling results from the Global Flood Monitoring System to analyze how floods affect the probability of anemia in Afghan women of reproductive age (15–49). In addition to establishing a causal relation between the two by exploiting the quasi-random variation of floods in different districts and periods, the analysis demonstrates that floods have a significant positive effect on the probability of anemia through two possible transmission mechanisms. The first is a significant effect on inflammation, probably related to water borne diseases carried by unsafe drinking water, and the second is a significant negative effect on retinol concentrations. Because the effect of floods on anemia remains significant even after we control for anemia’s most common causes, we argue that the condition may also be affected by elevated levels of psychological stress.

## 1 Introduction

As the most common type of disaster worldwide, floods, which account for 47% of all catastrophic events, resulted in approximately 57,000 deaths between 2006 and 2015 [1]. Future prospects are even bleaker: the frequency and intensity of floods are expected to increase because of global warming through rising sea levels and more extreme precipitation [2–5]. Exposure to floods will also increase because of rapid urbanization [6]. The effect of floods is particularly strong in Afghanistan, where they accounted for over 5,000 recorded deaths and about 400,000 displaced people between 1988 and 2006, over 200,000 of these in the 2002–2006 period alone [7]. In 2013, the data year for this study, floods accounted for 70% of all the natural disasters in Afghanistan [8].

The most evident effects of floods, besides loss of life and injuries, are crop losses, problems accessing markets, and depletion of household wealth, all of which ultimately determine food insecurity and related micronutrient deficiencies. According to one systematic review of 197 studies [6], floods also have significant and often lasting health consequences, including disability, social disruption, and mental health problems. These negative health outcomes are underscored by an earlier review of 212 epidemiologic studies evidencing their influence on common mental disorders, posttraumatic stress disorder (PTSD), and suicide [2], results which hold also in the long-run as shown in a study on early chilhood exposure to natural disasters and mental health disorders and substance use as adults [9]. The negative mental health effects of flooding caused by hurricanes Sandy and Katrina have also been documented [10, 11].

Floods may also explain the incidence of anemia (low hemoglobin) in Afghanistan, a major public health problem that, in 2013, affected 40.4% of reproductive age women, 13.8% of them suffering from iron deficiency anemia [12]. In fact, studies for Southeast Asia show not only that nearly every second pregnant women is anemic, but that this condition causes about 20% of all maternal deaths [13]. The most common form of the condition is iron deficiency anemia, which in Southeast Asia is caused primarily by inadequate intake of iron, dietary deficiency, and infectious diseases [13]. An additional but as yet little studied cause of such anemia could be severe psychological stress, already shown to heavily impact menstrual disorders [14–16], which in turn may lead to anemia. In this paper, therefore, we analyze the possible effects of floods on anemia among a particularly susceptible group: Afghan women of reproductive age.

Our primary hypothesis is that floods can affect anemia through a number of channels, including infections, poor diet (leading to iron, zinc, and vitamin deficiencies), and psychological stress associated with the consequences of flooding. In testing this hypothesis, we contribute to the existing literature in several ways: first, as emphasized by other studies (e.g., [6]), there is a dearth of research on the health consequences of floods in developing countries. This scarcity is particularly true for Afghanistan, a country for which, to the best of our knowledge, no research on this topic exists. Second, little of the existing research on the health effects of flooding focuses on reproductive age women even though this population is particularly vulnerable to natural disasters [17]. As pointed out in [17], “women’s elevated risk for worse mental health outcomes following disaster exposure, combined with negative impacts of prenatal stress on maternal health, calls for a greater attention to women’s reproductive health following flood events” (p. 46). Finally, this study can serve as a primer for combining rich biomarker information and satellite imagery to identify the causal relations between flood exposure and the prevalence of anemia.

## 2 Data and measures

### 2.1 Data sources

Information on the health and other relevant characteristics of reproductive age Afghan woman, together with household level information, are taken from the 2013 National Nutrition Survey (NNS) Afghanistan. The NNS (2013) was undertaken through a cooperative agreement between the Aga Khan University, Pakistan, the Afghan Ministry of Public Health and UNICEF Afghanistan. The NNS data are publicly available from UNICEF. The NNS collects data on the nutritional status of the Afghan population and related factors, such as consumption frequency of specific food groups during the week and month before interview and the unavailability of any food during the month prior to interview. The survey sample is obtained using a two-stage cluster sampling technique based on the Afghan Central Statistical Office (CSO) sampling frame and includes both urban and rural households.

In addition to general information on household characteristics—including measures related to wealth, food diversity and frequency, and anthropometric measures—the 2013 NNS provides biomarker data for children (0–59 months), unmarried adolescent girls (10–19), and married women of reproductive age (15–49). We restrict our sample to this latter group for whom the survey data record hemoglobin, ferritin, retinol, vitamin D, zinc, C-reactive protein (CRP), and alpha-1-acid glycoprotein (AGP) blood levels, as well as urinary iodine excretion. The serum and blood samples were collected by trained phlebotomists, placed in gel ice-packs and dry ice, and transported first to Kabul via airline cargo and then to Karachi [12]. Hemoglobin levels were tested on site using a HemoCue machine, thereby avoiding the (common) destruction of erythrocytes during blood sample transportation [12]. Although the Nutritional Research Laboratory of Aga Khan University also analyzed other biomarkers, the data set includes no information on the target group’s vitamin B12 (folic acid) levels, whose deficiency, together with that of iron and vitamin A, is the most common cause of anemia from inadequate food intake. We overcome this limitation by exploiting individual level information on the intake frequency of vitamin B12 rich food groups (meat/fish, dairy/eggs) in the week prior to interview.

Although the survey sample is provincially representative, because of budget limitations, the biomarker information was collected only for a nationally representative subsample. It should further be noted that the 2013 NNS is not seasonally representative in that data collection was carried out between the second week of June 2013 and the end of October 2013, with a 50-day suspension during Ramadan to avoid biases from changed eating behaviour during this observance. After deletion of missing and implausible values (observations showing serum ferritin > 150 *μ*g/dL, and CRP > 10 mg/dL are dropped from the final study sample), the final analytic sample comprises 1,128 observations, which decreases to 979 when we drop observations for which data on serum ferritin, retinol, zinc, and CRP are unavailable. The original sample included 1,237 married woman of reproductive age, 100 of them missing values for hemoglobin levels and 9 missing information on basic household characteristics.

The Global Flood Monitoring System (GFMS) database, a NASA-founded project developed at the University of Maryland, provides real-time (3-hour interval) quasi-global (50°N—50°S) flood detection and intensity estimates using TRMM Multi-satellite Precipitation Analysis (TMPA) precipitation information, hydrological runoff, and routing models at 0.125° longitude/latitude grid resolution. Routing is a widely used technique in hydrology that enables the prediction of changes in surface water state. Runoff routing is the process of routing excess rainfall to produce a hydrograph of accumulated surface water [18]. For each grid location, floods are reported in millimeters of accumulated water above the historical threshold derived from 13 years of retrospective statistics on surface water storage [19]. A flood is detected for each grid’s cell when *R* > *P*_95_ + 0.5 * *δ* and *Q* > 10, where *R* is the routed runoff in millimeters for each 3-hour interval. *P*_95_ is the 95th percentile value, *δ* is the temporal standard deviation of the routed runoff derived from the retrospective simulation time series at the grid cell, and *Q* is the corresponding value of discharge in m^3^/s [19]. Particularly relevant for the case of Afghanistan is the ability of GFMS’s cold season process to detect snowmelt-related floods, which, because of heavy winter snowfalls combined with warm summers, make up a large part of the country’s inundation [20].

### 2.2 Dependent variable: Anemia

The outcome of interest is the anemia status of married women of reproductive age (15–49), defined by the WHO threshold of hemoglobin concentration levels less than 12mg/dl (<11mg/dl for pregnant women) [21] and adjusted in the original data for altitude. We thus define a binary variable equal to 1 if low concentration is observed and 0 otherwise. To identify the channels through which floods affect anemia, we also regress a selection of characteristics known to influence anemia on flooding. In doing so, we use the following known causes of anemia as dependent variables: ferritin (*Fer*_*i*_ < 12*μ*g/dL), serum retinol (*Ret*_*i*_ < 20*μ*g/dL), current inflammation status (*CRP*_*i*_ > 1*mg*/*dL*), and availability of safe drinking water. This latter—defined as piped water; bottled water; water from hand pumps, protected springs, or wells; or water brought by tanker trucks—is a binary variable equal to 1 if the household has access to safe drinking water, 0 otherwise.

### 2.3 Flood variable

A main explanatory variable in this study is the district level daily average of accumulated land surface water in millimeters above the flood threshold measured one and two months before the interview date (*Flood*_*i*,*t*−*m*_). Its construction involves cropping each GFMS file based on Afghanistan’s national borders and then obtaining daily flood values for each grid cell by computing the average flood estimate for the 3-hour interval flood estimates (8 files per day). This process yields a georeferenced database of daily flood estimates whose values are assigned to each observation in the 2013 NNS based on the interview date and district of residence by projecting them onto the AGCHO shapefile for the second administrative division (districts) [22]. Although the spatial disaggregation level of the GFMS data is relatively high, flood estimates cannot be assigned to 9 of the 398 Afghan districts because of their relatively small area. Hence, to prevent missing values in the variable of interest, we increase the resolution from 0.125° to 0.0625° longitude/latitude by dividing each grid’s cell in the GFMS data by four, and assigning to these subcells the same flood estimate as given by the original corresponding cell. We also eliminate uninhabited areas from the flood variable estimations by exploiting the high spatial resolution (0.0083°) global population estimates from the Gridded Population of the World (GPWv4) 2010 raster image [23]. Lastly, we assure comparability of the one and two-month flood coefficients by computing these variables as daily averages; that is, we divide the count of floods in millimeters at *t-1* and *t-2* by 30.4 and 60.8 days, respectively. We then adjust the final measures for district population density (1,000 inh./100 km^2^). District population data are taken from CSO Population Statistics by Civil Division and Sex, 2013 [24]. Eq 1 expresses the flood measure construction for *t-1,t-2*, where *F*_*h*,*d*,*r*_ are 3-hour interval (*h*) flood estimates (in mm above the flood threshold) for all the grid’s cells corresponding to the district of residence (*r*_*i*_) of the *i*^*th*^ observation relative to a single day *(d)*, *int*_*i*_ is date of interview, *dens*_*r*,*i*_ is population density of district *r*_*i*_, and *m* = 1,2 is the time span in months.

$$Flood_{i,t}=\frac{\sum_{d=int_{i}-30*m}^{int_{i}}\sum_{h=1}^{8}\frac{F_{h,d,r_{i,}}}{8}}{30.4*m}\frac{1}{dens_{r_{i}}}$$ (1)

### 2.4 Biomarkers and nutrition characteristics

These characteristics are computed as a set of dummy variables equal to 1 for observations deficient in serum ferritin (*Fer*_*i*_ < 12 *μ*g/dL), serum retinol (*Ret*_*i*_ < 20*μ*g/dL), and serum zinc (*Zn*_*i*_ < 60*μg*/*dL*), or with a low intake frequency of vitamin B12/B9 rich food groups (meat/fish, dairy products) in the week before interview (*B*12/*B*9_*i*_ ≤ 7*days*), 0 otherwise. All thresholds are based primarily on WHO criteria [25–27]. The original data set provides ferritin, retinol, and zinc levels adjusted for inflammation status as measured by CRP and AGP. CRP, however, can be differentiated into a more severe (*CRP*_*i*_ > 3*mg*/*dL*) and a more moderate (*CRP*_*i*_ > 1*mg*/*dL*) condition. Although the WHO recommends the former [27], we adopt the latter—also used in the official UNICEF NNS report [12]—because it covers a more meaningful proportion of our sample. Lastly, to control for the possible effects on anemia of fasting during the month of Ramadan, we construct a dummy variable equal to 1 if an individual was interviewed after August 7, 2013, 0 otherwise.

### 2.5 Other relevant constructs

We further control for a set of women’s, household, and provincial characteristics, including age of respondent and household head in years, a dummy equal to 1 if a woman is currently pregnant (0 otherwise), dummies for the educational level of both the reproductive age women and corresponding household head equal to 1 if an individual is literate, 0 otherwise, and another equal to 1 if the household head is currently married, 0 otherwise. Although the raw data includes information about the highest educational level achieved, because of the extremely high illiteracy rate, especially among women (74%), we choose to measure only whether an individual can at least read and write. Additional controls at the household level are the dependency ratio, computed as the proportion of economically inactive members in the household (individuals aged 0–14 and over 65) divided by the number of active members (15–64); a set of eight dummy variables identifying ethnolinguistic group (Baluchi, Dari, Hazarai, Nuristani, Pashaee, Pashtu, Turkmeni, and Uzbeki), and the household wealth index. This latter is computed by a polychoric principal component analysis applied to a set of relevant characteristics; namely, household dwelling building material; and dummy variables for ownership of bicycle, motorcycle, car, television, telephone, mobile phone, sewing and washing machines, and livestock, as well as availability of electricity and safe water in the dwelling [28]. Note that in models where safe drinking water is used as the dependent variable, this variable is excluded from the computation of the wealth index. Safe drinking water is a dichotomous variable equal to 1 if the household has access to the protected drinking water sources enumerated earlier, 0 otherwise. Finally, we control for committed aid at the provincial level for 2013 (in 100 thousand USD) by exploiting information from the Afghan National Budget and Aid Management Systems [29].

## 3 Empirical model

The study aims to identify the causal relations among the occurrence and intensity of floods and anemia in reproductive age Afghan women by exploiting the quasi-random flood variation among different districts and periods. We estimate this effect by means of the following linear probability model:

$$Y_{i}=\alpha +\beta_{1}Flood_{i,t}+\beta_{2}Aid_{p,}+\gamma_{w}CW_{i}+\gamma_{h}CH_{i}+\gamma_{p}P+\varepsilon_{i}$$ (2)

where *Y*_*i*_ is a binary variable equal to 1 if the *i*^*th*^ woman’s hemoglobin concentration is less than 12mg/dL (< 11mg/dL for pregnant women), 0 otherwise. The controls are a set of woman (***CW***) and household characteristics (***CH***), namely woman’s age, literacy, and current pregnancy status; household location type (urban vs. rural), wealth index, dependency ratio, ethnolinguistic group; and age, sex, literacy, and marital status of the household head. We also control for province fixed effects (***P***) and provincial committed aid (*Aid*_*p*,_). *ε*_*i*_ is an idiosyncratic error term.

$$Y_{i}=\alpha +\beta_{1}Flood_{i,t}+\beta_{2}Aid_{p}+\gamma_{b}BNC_{i}+\gamma_{w}CW_{i}++\gamma_{h}CH_{i}+\gamma_{p}P+\varepsilon_{i}$$ (3)

Eq (3) models the causal relation between floods and anemia status while directly controlling for a vector of the following biomarkers and nutrition characteristics (***BNC***): serum ferritin (*Fer*_*i*_), serum retinol (*Ret*_*i*_), and serum zinc (*Zn*_*i*_) deficiencies; inflammation status (*CRP*_*i*_); and meat, fish, and dairy product intake frequencies in the week before interview (*B*12/*B*9_*i*_). This specification allows us to determine which biomarkers and nutritional characteristics are associated with anemia in our sample, and also to what extent the inclusion of these variables attenuates the flood effect on anemia. Once we identify the former, we use the same estimation strategy as in Eq (2) to regress the significant characteristics on floods to determine the mechanisms through which floods affect anemia.

For this analysis, linear probability models are preferable to their nonlinear probit or logit counterparts [30] for three methodological reasons. First, because of its design, the NNS (2013) tends to within-cluster similarities that may bias the coefficients’ standard errors. We overcome this shortcoming by computing robust standard errors clustered at the (53) district levels. At the same time, in the presence of heteroscedasticity, the maximum likelihood estimator is inconsistent, meaning that its point estimates are biased even when computing a robust covariance matrix [31] (pp. 692–693). Linear models, in contrast, provide consistent estimates despite heteroscedasticity of the error term, while also yielding easily interpretable coefficients.

Although occurrence and intensity of floods could be assumed to be quasi-random, there is the possibility that some degree of correlation exists between these and human-made disasters. In particular, in a country such as Afghanistan, the possible role of conflict as a confounder must be considered when establishing causality for the floods-health nexus. In this respect, using data from Uppsala Conflcit Data Program Georeferenced Database (version 17.1) [32] we computed a set of district level measures of conflict intensity for the same time span for which the floods variables are computed (i.e. one and two months before the interview). Conflict intensity is measured as the total number of per capita fatalities. Moreover, a similar argument could be raised in respect to the environmental characteristics in which the households live. In fact, as shown by extant literature (e.g., [33]), urbanization is a direct determinant of the occurence and intensity of floods.

Correlation analysis (Table 1) shows that neither our flood measures nor population density are significantly correlated with conflict intensity, ruling out the possibility that omitting these variables from the set of controls causes bias in the estimates of interest. It should be noted that the dicothomus variable controlling for households living in rural vs. urban locations is negatively correlated with flood, confirming that these natural disasters are more likely to happen in urbanized areas. Moreover, we anticipate that using these variables as additional controls does not change either the size of the flood’s coefficients or their statistical significance in all models. However, due to the possible endogeneity of the conflict variable (e.g., we find that greater levels of conflict decreases the probability of anemia), we report only the models excluding this control. These additional results are available from the authors upon request.

**Table 1:** Measures of flood, conflict and urbanization: Correlation analysis.

	Flood_t−1_ (pop)	Flood_t−1_ (den)	Flood_t−2_ (pop)	Flood_t−2_ (den)
	(1)	(2)	(3)	(4)
				
Fat_t−1_ (pop)	0.019	-0.009	–	–
	(0.523)	(0.768)		
				
Fat_t−2_ (pop)	–	–	0.038	-0.006
			(0.195)	(0.837)
				
Pop. Density	-0.025	-0.023	-0.037	-0.029
	(0.400)	(0.425)	(0-214)	(0.327)
				
Rural (dummy)	-0.135	-0.140	-0.086	-0.121
	(0.000)	(0.000)	(0.000)	(0.000)

*Notes*: Table reports Pearson correlation coefficients for all variables with the exception of the rural dummy for which tetrachoric correlations coefficients are shown. P-values in parenthesis are computed using a 95% confidence interval.

Finally, we argue that although floods may be sometimes predictable and thus households could move away from their habitual residence, these latter are unlikely to migrate outside their district of residence when a flood occurs. However, even in presence of such an error in measuring our flood variable we would underestimate the true negative effect of floods on anemia.

## 4 Results

A comparison of the descriptive statistics for the full versus the reduced sample (because of missing biomarker values) reveals no abnormal divergence (Table 2). In fact, the absence of statistically significant differences in variable means (column 5) suggests that the unavailability is completely random. This assumption is further confirmed by the fact that the total number of districts in the reduced sample does not change. In general, the descriptive statistics indicate that 43% of reproductive age Afghan women suffer moderate anemia. Only 26% of those in the sample (average age 30.4 years) are literate, as opposed to 48% of the household heads. Most of the women (91%) are married, with the rest being widows (8%) or separated (1%). The reproductive age female population also tends to suffer from micronutrient deficiencies, with 30%, 13%, and 21% being deficient in iron, vitamin A, and zinc, respectively. In half the sample, the intake frequency of meat/fish or dairy/eggs is less than seven times per week. About 4% of the sample has a *CRP*_*i*_ > 1*mg*/*dL* with only 0.7% having a *CRP*_*i*_ > 3*mg*/*dL*. Finally, 18% and 32% of the reproductive age women experienced at least one day of flooding during *t-1* and *t-2*, respectively.

**Table 2:** Descriptive statistics.

	Mean	SD	Mean	SD	Difference (p value)
	(1)	(2)	(3)	(4)	(5)
					
Anemia^a^	0.43	0.50	0.43	0.50	0.499
Age	30.36	7.97	30.29	7.87	0.417
Woman is literate^a^	0.26	0.44	0.26	0.44	0.451
Woman is pregnant^a^	0.13	0.33	0.14	0.34	0.755
Interviewed after Ramadan^a^	0.35	0.47	0.37	0.48	0.732
Wealth index^b^	0.00	1.25	-0.02	1.25	0.362
Dependency ratio^c^	1.27	0.83	1.30	0.83	0.741
Safe water^a^	0.59	0.49	0.58	0.49	0.468
Head is literate^a^	0.48	0.50	0.47	0.50	0.478
Head age	45.12	14.51	44.94	14.67	0.393
Head is married^a^	0.91	0.28	0.91	0.28	0.477
Flood: 1 month (mm)^d^	0.31	1.68	0.32	1.70	0.568
Flood: 2 months (mm)^d^	1.68	4.38	1.63	4.28	0.399
Flood: 1 month^a^	0.18	0.38	0.18	0.38	0.576
Flood: 2 months^a^	0.32	0.47	0.33	0.47	0.652
Iron deficiency (*Fer*_*i*_ < 12 *μ*g/dL)^a^			0.30	0.46	
B12 low intake (*B*12/*B*9_*i*_ ≤ 7*days*)^a^			0.51	0.50	
Vit. A deficiency (*Ret*_*i*_ < 20*μg*/*dL*)^a^			0.13	0.34	
Zinc deficiency (*Zn*_*i*_ < 60*μg*/*dL*)^a^			0.21	0.41	
Inflammation (*CRP*_*i*_ > 1*mg*/*dL*)^a^			0.04	0.21	
Inflammation (*CRP*_*i*_ > 3*mg*/*dL*)^a^			0.007	0.08	
Number of districts	53		53		
Number of observations	1128		979		

*Notes*: Column (5) is calculated using *t*-tests.

^a^Dummy variables.

^b^Computed by a polychoric principal component analysis applied to a set of assets, as well as the availability of electricity and safe water.

^c^The proportion of economically inactive members in the household (individuals aged 0-14, and over 65) divided by the number of active members (15–64).

^d^The district level daily average count of accumulated land surface water in millimeters above the flood threshold measured at one and two months before the interview date.

**Fig 1:**
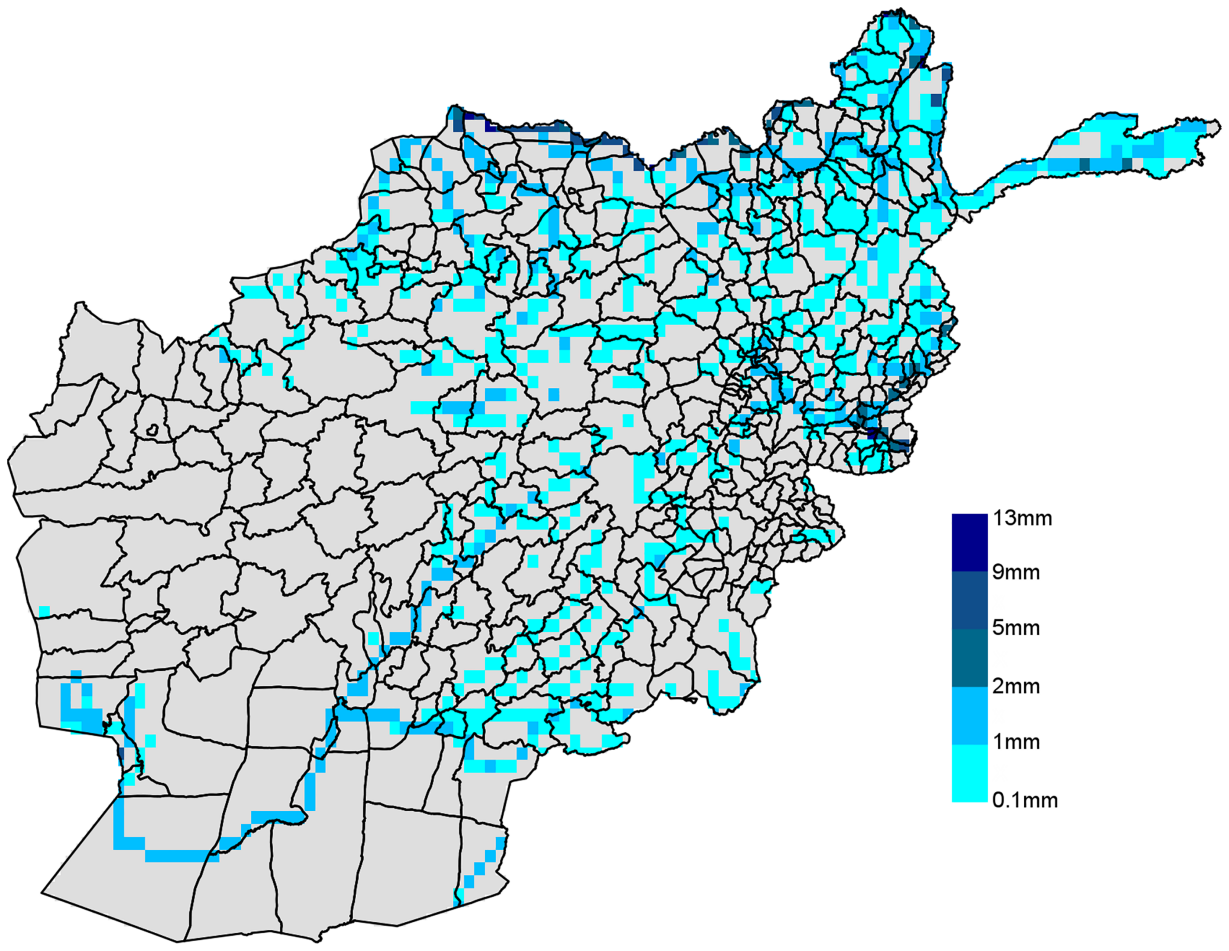
Daily average floods in millimetres (April-October 2013). Drawn by the authors using GFMS data. Resolution 0.125° longitude/latitude.

The spatial variation in flood intensity among the districts is outlined in Fig 1. A considerable area of the country has been affected by floods in 2013. This is also highlighted by the fact that 32% of the observations in our sample experienced at least a day of flooding in the two months before the interview. Most of the floods occurred in the south-east and north-east of the country. Flood-prone areas are located close to large rivers, such as the Amu Darya, a major river in Central Asia that marks the border between Afghanistan and Tajikistan, and the Helmand River in the south-west. Floods have been particularly intense in the area south-west of the Kabul province and north of Kunduz where values between 1 and 5 mm of flood per day have been recorded.

Table 3 reports the coefficients of interest for the restricted and full models employing a binary dependent variable that captures whether or not a woman is anemic (hemoglobin concentration < 12mg/dL; < 11mg/dL for pregnant women). Here, the coefficients capturing floods are always statistically significant, and we observe no abnormal changes in magnitude among different specifications (see Table A in S1 File for complete model estimates and models using floods adjusted by 1,000 inh.). Specifications 1 and 2 (Eq 2) show the effect of floods occurring one and two months before the interview, measured in millimeters per day adjusted by district population density. All else kept constant, one extra millimeter of flood per 1,000 inhabitants per 100 km^2^ increases the probability of anemia by 0.039 and 0.054 at *t-1* and *t-2*, respectively.

**Table 3:** Effect of floods on anemia: OLS estimates.

	*Dependent variable*			
	Anemia			
	(1)	(2)	(3)	(4)
				
Flood_*t*−1_	0.039***		0.024***	
	(0.006)		(0.009)	
Flood_*t*−2_		0.054***		0.033***
		(0.009)		(0.013)
Iron deficiency			0.219***	0.219***
			(0.037)	(0.037)
Inflammation (*CRP*_*i*_ > 1*mg*/*dL*)			0.129	0.129
			(0.099)	(0.099)
Vit. A deficiency			0.202***	0.202***
			(0.062)	(0.062)
Zinc deficiency			0.009	0.009
			(0.070)	(0.070)
Vit. B12 low intake			0.043	0.043
			(0.048)	(0.048)
Province fixed effects	Yes	Yes	Yes	Yes
Ethnicity fixed effects	Yes	Yes	Yes	Yes
				
Observations	1,128	1,128	979	979
*R*^2^	0.084	0.084	0.139	0.139
Adjusted *R*^2^	0.048	0.048	0.096	0.096
Residual std. error	9.384	9.384	9.007	9.007
*F* statistic	2.356***	2.355***	3.202***	3.201***

*Notes*: The dependent variable is equal to 1 if the respondent’s hemoglobin concentration is less than 12mg/dL (< 11mg/dL for pregnant women) and 0 otherwise. The regressions control for woman’s age, literacy, and current pregnancy status; household location type (urban vs. rural), wealth index, dependency ratio, and ethnolinguistic affiliation; and age, sex, literacy, and marital status of household head, while also including provincial dummies and provincial aid. All models are estimated using sampling survey weights, and the flood variable in all specifications is adjusted for district population density. Robust standard errors (in parenthesis) are clustered at the district level.

* *p* < 0.1,

** *p* < 0.05,

*** *p* < 0.01.

Table 3 also presents the results of applying specifications 3 and 4 (Eq 3) to the full model. Here, the inclusion of the ***BNC*** vector variables increases the goodness-of-fit, from an adjusted *R*-squared of 0.048 in the restricted model to a value of 0.096 in the full model. As expected, iron and vitamin A deficiency increase the probability of anemia by a substantial 0.22 and 0.20, respectively, but we identify no statistically significant effect of inflammation, zinc deficiency, or low frequency intake of vitamin B12 rich foods (< 7 times/week) on the probability of becoming anemic. If we use the more stringent WHO definition of inflammation (*CRP*_*i*_ > 3*mg*/*dL*) [27], however, then we observe a positive association between this latter and inflammation (see Table B in S1 File). Thus, severe inflammation is significantly related to anemia in our sample but moderate inflammation is not. Lastly, despite directly controlling for the most common causes of anemia that are likely to be correlated with floods, the coefficients of interest remain statistically significant, although smaller in magnitude than their restricted model counterparts (0.23 and 0.33 for *t-1* and *t-2*, respectively). These results indicate that floods may affect anemia through mechanisms other than nutritional deficiencies and inflammation conditions (e.g., floods may cause psychological stress, which in turn may cause prolonged and abnormal uterine bleeding among women of reproductive age) or that our variables are subject to a certain degree of measurement error (especially given our inability to account directly for folate levels).

Table 4 then depicts the effect of floods on levels of serum ferritin (regressions 1 and 2) and serum retinol (regressions 3 and 4) measured in *μ*g/dL, revealing no statistically significant flood effects on the former. One plausible explanation is that only a small proportion of households suffered severe hunger in the month before the interview (0.08%). We derive this result by computing the Hunger Scale Index for our sample [34]. This result suggests that households exposed to floods are able to maintain a constant consumption of cereals, particularly wheat, which is not only the main food staple in Afghanistan but also the main source of iron [35]. In fact, Afghan wheat varieties are rich in iron [36], concentrations range between 5.5 and 12.2mg/100gr, as compared with a recommended daily intake of iron of 30mg and 60mg for pregnant women [37]. The intake of vitamin A rich foods (i.e., fresh fruit and vegetables, and food from animal sources) may also diminish as a consequence of natural disasters like floods: in regressions 3 and 4, all else being equal, one extra millimeter per day of flooding at t-1 and t-2 causes decreases in serum retinol of 2.2 and 3.2*μ*g/dL, respectively (Table 4).

**Table 4:** Effect of floods on serum ferritin and serum retinol: OLS estimates.

	*Dependent variable*			
	Ferritin (*μ*g/dL)		Retinol (*μ*g/dL)	
	(1)	(2)	(3)	(4)
				
Flood_t−1_	-0.544		-2.209***	
	(2.934)		(0.383)	
Flood_t−2_		-0.735		-3.208***
		(4.283)		(0.543)
Province fixed effects	Yes	Yes	Yes	Yes
Ethnicity fixed effects	Yes	Yes	Yes	Yes
				
Observations	979	979	979	979
*R*^2^	0.097	0.097	0.099	0.099
Adjusted *R*^2^	0.054	0.054	0.057	0.058
Residual std. error	577.950	577.951	240.588	240.581
*F* statistic	2.271***	2.270***	2.386***	2.388***

*Notes*: In regressions 1 and 2, the dependent variable is level of serum ferritin in *μ*g/dL, whereas in regressions 3 and 4, it is serum retinol in *μ*g/dL. These regressions control for woman’s age, literacy, and current pregnancy status; household location type (urban vs. rural), wealth index, dependency ratio, ethnolinguistic affiliation; and age, sex, literacy, and marital status of household head, while also including provincial dummies and provincial aid. Only for regressions 1 and 2 is inflammation status (CRP > 1 mg/dL) used as a regressor. All models are estimated using sampling survey weights, and the flood variable in all specifications is adjusted for district population density. Robust standard errors (in parenthesis) are clustered at the district level.

* *p* < 0.1,

** *p* < 0.05,

*** *p* < 0.01.

A major reason for floods being a known cause of infectious diseases is impaired household access to safe drinking water [17]. Table 5 summarizes the causal relations between floods and inflammation status—as measured by elevated concentrations in the blood of CRP (1*mg*/*dL*)—and between floods and the availability to the household of safe water. Regressions 1 and 2 show that, all else being equal, an extra millimeter per day of flood at *t-1* and *t-2* causes a 0.015 and 0.023 increase, respectively, in the probability of inflammatory conditions, severe cases of which are associated with anemia (Table B in S1 File). Floods also greatly reduce the probability of access to safe drinking water: a 0.12 and 0.18 decrease for floods measured at *t-1* and *t-2*, respectively.

**Table 5:** Effect of floods on inflammation and availability of safe water: OLS estimates.

	*Dependent variable*			
	Inflammation		Safe water	
	(1)	(2)	(3)	(4)
Flood_t−1_	0.015***		-0.124***	
	(0.005)		0.035	
Flood_t−2_		0.023***		-0.179***
		(0.006)		(0.050)
Province fixed effects	Yes	Yes	Yes	Yes
Ethnicity fixed effects	Yes	Yes	Yes	Yes
				
Observations	979	979	889	889
*R*^2^	0.063	0.064	0.289	0.289
Adjusted *R*^2^	0.021	0.021	0.256	0.256
Residual std. error	4.352	4.351	7.550	7.550
*F* statistic	1.511**	1.511**	8.624***	8.625***

*Notes*:In regressions 1 and 2, the dependent variable is inflammation status (CRP > 1 mg/dL), whereas in regressions 3 and 4, it is access to safe water (1 if yes, 0 otherwise). These regressions control for woman’s age; household location type (urban vs. rural), wealth index, dependency ratio, and ethnolinguistic affiliation; and age, sex, literacy, and marital status of household head, while also including provincial dummies and provincial aid. Only specifications 1 and 2 also control for woman’s current pregnancy status. All models are estimated using sampling survey weights, and the flood variable in all specifications is adjusted for district population density. Robust standard errors (in parenthesis) are clustered at the district level.

* *p* < 0.1,

** *p* < 0.05,

*** *p* < 0.01.

Lastly, we consider additional covariates (reported for all models in Tables A-C in S1 File), including a flood variable adjusted for population in 1,000 inh. The models using this latter (regressons 5–8 in Table A in S1 File) confirm the main results presented in Table 5. In all regressions, we note that younger women are less likely to be anemic (Tables A and B in S1 File) while also having higher levels of serum ferritin (regressions 1–2, Table C in S1 File). We find no statistically significant effect of female literacy on any of the dependent variables, either because very low education levels do not actually affect health outcomes or because this effect is too small to observe. As all models in Table A in S1 File show, being pregnant increases the probability of low hemoglobin levels and has a negative effect on serum ferritin and serum retinol levels (regressions 1–4, Table C in S1 File). Interestingly, we observe a strong increase in the anemia probability for women interviewed after Ramadan. Although unable to determine whether or not a woman fasted during this period, we can safely assume that the post-Ramadan interview is random in both observable and nonobservable characteristics, meaning that our estimates are not biased by any systematic error. Anemia is also more likely among women in rural households, who on average have a lower level of serum retinol. The positive association between household wealth and anemia probability, however, cannot be taken at face value because it is influenced by the wealth variable’s correlation with other model covariates, particularly the literacy status of both the woman and the head of household. When these variables are dropped, the wealth coefficient becomes statistically insignificant.

## 5 Discussion

The empirical research on flooding’s effect on health in developing countries is limited [6], and particularly so for Afghanistan, where floods have been a common occurrence in the past decade, claiming thousands of lives and displacing many more. In fact, about one third of our representative sample of reproductive age women experienced a flood in the two-month period prior to the survey, leading us to argue that these floods are a cause of anemia among this population. We test this proposition by using GFMS satellite imagery to accurately capture flood locations and intensity, and by exploiting the quasi-random variation of floods among different districts and time periods to capture their causal relation with the incidence of anemia.

Our results show that the flood effect on anemia is not only significant but also quite large: an extra millimeter per day of population density-adjusted flooding increases anemia probability by 0.039–0.054. Floods influence anemia via several channels but particularly by reducing the intake of such vital nutrients as iron, zinc and, vitamins through the destruction of crops and livestock. They also have a well-documented effect on water borne diseases—including gastrointestinal and respiratory diseases, skin infections, and leptospirosis [17]—which can also result in anemia. Lastly, given the damage that floods wreak on lives and livelihoods (especially in developing countries like Afghanistan where insurance is rare), they generate psychological stress in those affected [17] that itself can cause a decrease in serum iron and/or affect erythropoiesis [38]. Psychological stress can also negatively affect menstrual cycles [14–16], which in turn may result in anemia.

Our analysis identifies two main transmission mechanisms of floods on anemia: inflammation or water-related infections and vitamin A intake. As regards the first, not only is anemia clearly associated with severe inflammation but an extra millimeter per day of floods in the previous two months induces a 2.3% increase in the probability of moderate inflammation. At the same time, floods’ negative affect on the availability of potable water is often an important cause of infections related to water-borne diseases. Flooding’s negative impact on the intake of vitamin A rich foods (i.e., fresh fruit and vegetables) is reflected by the fact that one extra millimeter per day of flooding in the previous two months decreases serum retinol by 3.2 *μ*g/dL. Flooding does not, however, appear to influence ferritin intake, which probably implies that floods have no strong effect on the consumption of wheat, which is the primary iron rich staple in the Afghan diet. This result suggests that, in the case of floods, vitamin A supplementation may be more needed than iron, at least for Afghan reproductive age women.

Although biomarker information and nutrition characteristics can partly explain the probability of anemia, the effect of floods on this condition remains significant even after we include these variables in our regressions. This interesting finding indicates that floods may be affecting anemia beyond the most common determinants of inadequate iron intake, dietary deficiency, and infectious diseases. The most obvious explanation for our sample of reproductive age Afghan women is that floods, through their effect on stress, affect menstrual cycles, including prolonged and abnormal uterine bleeding. Admittedly, our data set provides no information with which to test this assumption—although the relation is empirically supported by prior research—however, given the breadth of our explanatory analytic variables, it seems a plausible explanation.

Two other study limitations include the fact that although our flood exposure measure is known for its high precision, the unavailability of household GPS coordinates limits its accuracy in our case. Knowing the exact location of a household, and, in particular, the household’s proximity to the flood, would be useful as previous research has shown that mental health problems are predominantly clustered in regions that are geographically more exposed to a natural disaster [11]. Likewise, the absence from the NNS (2013) of data on folate levels prevents our directly controlling for this vitamin, especially when interpreting the persistent statistically significant effect of floods after main transmission channels are controlled for.

## 6 Conclusions

Because both floods and anemia are serious and concomitant problems in South East Asia, and particularly in Afghanistan, this study assumes a relation between the two. We test this assumption using 2013 NNS data, which includes rich biomarker information, combined with GFMS satellite imagery that accurately captures the incidence of floods. Not only do our results demonstrate that floods are associated with anemia among Afghan women of reproductive age but, because of the quasi-randomness of flood occurrence, they can be interpreted as causal. These findings fill the knowledge gap left by a prior research focus on floods’ negative effects on health with no specific attention to their impact on anemia.

Our analysis identifies two channels through which floods influence anemia, the first being a significant effect on inflammation and a possible effect, through unsafe drinking water, on infections related to water-borne diseases. This latter is important because it underscores that anemia can be influenced by flood-related infections. Floods also significantly and negatively affect retinol concentrations, a recognized cause of anemia. Even with the inclusion of effective controls for anemia’s most common causes, our analysis still shows that floods affect anemia. Hence, given the empirical evidence of both flooding’s inducement of psychological stress and such the effect of stress on anemia, we speculate that this effect may be (at least partly) attributable to psychological stress.

## Supporting information

S1 File (**Table A**) Effect of floods on anemia: OLS estimates. (**Table B**) Effect of floods on anemia using the WHO inflammation definition: OLS estimates. (**Table C**) Effect of floods on drivers of anemia: OLS estimates.
(PDF)
