# Supplementary material for: The effect of floods on anemia among reproductive age women in Afghanistan
Source: PLoS One. 2018 Feb 9;13(2):e0191726. doi: 10.1371/journal.pone.0191726 (PMC5806855; doi:10.1371/journal.pone.0191726)
Supplement: S1 File — (Table A) Effect of floods on anemia: OLS estimates. (Table B) Effect of floods on anemia using the WHO inflammation definition: OLS estimates. (Table C) Effect of floods on drivers of anemia: OLS estimates. (PDF) [file pone.0191726.s001.pdf]

Supporting Information

Table A. Effect of floods on anemia: OLS estimates

	Dependent variable							
	Anemia							
	(1)	(2)	(3)	(4)	(5)	(6)	(7)	(8)
$Flood_{t-1}$	0.039***		0.024***		0.054***		0.033**	
	0.006		0.009		0.012		0.015	
$Flood_{t-2}$		0.054***		0.033***		0.066**		0.036
		0.009		0.013		0.027		0.028
Iron deficiency			0.219***	0.219***			0.219***	0.219***
			0.037	0.037			0.037	0.037
Inflammation ($CRP_i > 1mg/dL$)			0.129	0.129			0.129	0.129
			0.099	0.099			0.099	0.099
Vit. A deficiency			0.202***	0.202***			0.202***	0.202***
			0.062	0.062			0.062	0.062
Zinc deficiency			0.009	0.009			0.009	0.009
			0.070	0.070			0.070	0.070
ì Vit. B12 low intake			0.043	0.043			0.043	0.043
			0.048	0.048			0.048	0.048
Age	-0.005**	-0.005**	-0.004*	-0.004*	-0.005**	-0.005**	-0.004*	-0.004*
	0.002	0.002	0.002	0.002	0.002	0.002	0.002	0.002
Woman is literate	-0.042	-0.042	-0.048	-0.048	-0.042	-0.043	-0.048	-0.049
	0.045	0.045	0.055	0.055	0.045	0.045	0.055	0.055
Woman is pregnant	0.102*	0.102*	0.079	0.079	0.102*	0.102*	0.079	0.079
	0.055	0.055	0.055	0.055	0.055	0.055	0.055	0.055
Interviewed after Ramadan	0.120***	0.120***	0.107***	0.107***	0.120***	0.120***	0.107***	0.107***
	0.034	0.034	0.041	0.041	0.034	0.034	0.041	0.041
Urban	-0.168***	-0.167***	-0.164***	-0.164***	-0.167***	-0.167***	-0.164***	-0.163***
	0.036	0.036	0.054	0.054	0.036	0.036	0.054	0.054
Wealth index	0.031**	0.031**	0.032**	0.032**	0.031**	0.031**	0.032**	0.032**
	0.014	0.014	0.016	0.016	0.014	0.014	0.016	0.016
Dependency ratio	0.005	0.005	0.006	0.006	0.005	0.005	0.005	0.005
	0.016	0.016	0.019	0.019	0.016	0.016	0.019	0.019
Head is literate	-0.022	-0.022	-0.017	-0.017	-0.022	-0.022	-0.017	-0.017
	0.029	0.029	0.036	0.036	0.029	0.029	0.036	0.036
Head age	-0.001	-0.001	-0.0001	-0.0001	-0.001	-0.001	-0.0001	-0.0001
	0.001	0.001	0.002	0.002	0.001	0.001	0.002	0.002
Head is male	-0.034	-0.033	0.006	0.006	-0.034	-0.034	0.006	0.006
	0.111	0.111	0.103	0.103	0.111	0.111	0.103	0.103
Head is married	0.114	0.114	0.163*	0.163*	0.114	0.114	0.163*	0.163*
	0.093	0.093	0.089	0.089	0.093	0.093	0.089	0.089
Provincial aid	-0.043**	-0.041**	-0.04*	-0.039*	-0.042**	-0.04**	-0.039*	-0.038*
	0.018	0.018	0.021	0.022	0.018	0.018	0.022	0.021
Constant	2.009***	1.973***	1.612**	1.590**	2.000***	1.917***	1.606**	1.562**
	0.611	0.611	0.790	0.788	0.612	0.613	0.790	0.788
Ethnicity fixed effects	Yes	Yes	Yes	Yes	Yes	Yes	Yes	Yes
Province fixed effects	Yes	Yes	Yes	Yes	Yes	Yes	Yes	Yes
Observations	1,128	1,128	979	979	1,128	1,128	979	979
R^2	0.084	0.084	0.139	0.139	0.084	0.083	0.139	0.139
Adjusted R^2	0.048	0.048	0.096	0.096	0.048	0.048	0.096	0.096
Residual std. error	9.384	9.384	9.007	9.007	9.385	9.385	9.007	9.007
F statistic	2.356***	2.355***	3.202***	3.201***	2.354***	2.352***	3.201***	3.200***

Notes: The dependent variable is equal to 1 if the respondent's hemoglobin concentration is less than 12mg/dL (< 11mg/dL for pregnant women) and 0 otherwise. The regressions control for woman's age, literacy, and current pregnancy status; household location type (urban vs. rural), wealth index, dependency ratio, and ethnolinguistic affiliation; and age, sex, literacy, and marital status of household head, while also including provincial dummies and provincial aid. All models are estimated using sampling survey weights. The flood variable is adjusted for district population density in specifications (1)-(4), and for district population only in specifications (5)-(8). Robust standard errors (in parenthesis) are clustered at the district level.* $p < 0.1$, ** $p < 0.05$, *** $p < 0.01$.

Table B. Effect of floods on anemia using the WHO inflammation definition: OLS estimates

	Dependent variable	
	Anemia	
	(1)	(2)
Flood_{t-1}	0.028*	
	(0.009)	
Flood_{t-2}		0.039***
		(0.013)
Iron deficiency	0.226***	0.226***
	(0.037)	(0.036)
Inflammation ($CRP_i > 3mg/dL$)	0.483***	0.483***
	(0.130)	(0.130)
Vit. A deficiency	0.183***	0.182***
	(0.062)	(0.061)
Zinc deficiency	0.004	0.004
	(0.070)	(0.066)
Vit. B12 low intake	0.039	0.039
	(0.048)	(0.044)
Age	-0.004*	-0.004
	(0.002)	(0.003)
Woman is literate	-0.042	-0.042
	(0.055)	(0.055)
Woman is pregnant	0.077	0.077
	(0.055)	(0.055)
Interviewed after Ramadan	0.105**	0.105**
	(0.041)	(0.041)
Urban	-0.163***	-0.163***
	(0.054)	(0.054)
Wealth index	0.030*	0.030*
	(0.016)	(0.016)
Dependency ratio	0.0001	0.0001
	(0.0002)	(0.0002)
Head is literate	-0.020	-0.020
	(0.036)	(0.036)
Head age	0.0003	0.0003
	(0.002)	(0.001)
Head is male	-0.020	-0.020
	(0.103)	(0.107)
Head is married	0.209**	0.209**
	(0.089)	(0.103)
Provincial aid	-0.047**	-0.046**
	(0.020)	(0.020)
Constant	1.795**	1.769**
	(0.790)	(0.729)
Ethnicity fixed effects	Yes	Yes
Province fixed effects	Yes	Yes
Observations	979	979
R^2	0.149	0.149
Adjusted R^2	0.106	0.106
Residual std. error	8.953	8.953
F statistic	3.477***	3.477***

Notes: The dependent variable is equal to 1 if the respondent's hemoglobin concentration is less than 12mg/dl (< 11mg/dl for pregnant women) and 0 otherwise. All models are estimated using sampling survey weights, and all specifications use a flood variable adjusted for district population density. Robust standard errors (in parenthesis) are clustered at the district level. * $p < 0.1$, ** $p < 0.05$, *** $p < 0.01$.

Table C. Effect of floods on drivers of anemia: OLS estimates

	Dependent variable							
	Ferritin (µg/dL)		*Retinol (µg/dL)*		*Inflammation*		*Safe water*	
	(1)	(2)	(3)	(4)	(5)	(6)	(7)	(8)
$Flood_{t-1}$	-0.544		-2.209***		0.015***		-0.124***	
	(2.934)		(0.383)		(0.005)		(0.035)	
$Flood_{t-2}$		-0.735		-3.208***		0.023***		-0.179***
		(4.283)		(0.543)		(0.006)		(0.050)
Inflammation	0.438	0.438	2.045	2.047				
	(3.789)	(3.789)	(2.576)	(2.576)				
Age	0.464*	0.464*	0.036	0.036	0.001	0.001	-0.0001	-0.0001
	(0.239)	(0.239)	(0.077)	(0.077)	(0.002)	(0.002)	(0.002)	(0.002)
Woman is literate	-3.229	-3.228	1.534	1.536	0.034	0.034	-0.103*	-0.103*
	(4.295)	(4.295)	(1.732)	(1.731)	(0.024)	(0.024)	(0.055)	(0.055)
Woman is pregnant	-5.900**	-5.901**	-5.101***	-5.101***	0.043	0.043		
	(2.292)	(2.292)	(1.637)	(1.637)	(0.038)	(0.038)		
Interviewed after Ramadan	-9.775***	-9.773***	-2.425*	-2.421*	-0.007	-0.007		
	(3.356)	(3.357)	(1.249)	(1.251)	(0.013)	(0.013)		
Urban	-0.247	-0.257	-2.287*	-2.294*	0.014	0.014	-0.054	-0.054
	(6.784)	(6.804)	(1.294)	(1.292)	(0.016)	(0.016)	(0.061)	(0.061)
Wealth index	-1.323	-1.323	0.063	0.061	0.005	0.005	0.040*	0.040*
	(2.054)	(2.054)	(0.636)	(0.636)	(0.006)	(0.006)	(0.024)	(0.024)
Dependency ratio	0.544	0.544	1.360	1.361	-0.038***	-0.038***	0.006	0.006
	(1.155)	(1.155)	(0.912)	(0.912)	(0.013)	(0.014)	(0.029)	(0.029)
Head literate	-5.028	-5.029	1.561	1.561	0.021	0.021	0.154***	0.154***
	(3.439)	(3.439)	(1.322)	(1.323)	(0.017)	(0.017)	(0.053)	(0.053)
Head age	0.112*	0.112*	0.018	0.018	-0.001	-0.001	0.001	0.001
	(0.064)	(0.064)	(0.028)	(0.028)	(0.001)	(0.001)	(0.001)	(0.001)
Head is male	14.016***	14.013***	-2.836	-2.839	0.091	0.091	-0.075	-0.075
	(5.031)	(5.028)	(4.220)	(4.221)	(0.086)	(0.086)	(0.092)	(0.092)
Head is married	-8.336	-8.334	4.368	4.371	-0.122	-0.122	-0.031	-0.030
	(5.117)	(5.114)	(4.458)	(4.458)	(0.102)	(0.102)	(0.058)	(0.058)
Provincial aid	1.699	1.711	0.632*	0.685**	0.002	0.002	0.0001***	0.0001***
	(1.237)	(1.250)	(0.326)	(0.326)	(0.006)	(0.006)	(0.00002)	(0.00000)
Constant	-18.592	-18.838	8.694	7.602	0.117	0.125	-1.340***	-1.400***
	(26.308)	(26.625)	(6.544)	(6.534)	(0.187)	(0.186)	(0.520)	(0.521)
Ethnicity fixed effects	Yes	Yes	Yes	Yes	Yes	Yes	Yes	Yes
Province fixed effects	Yes	Yes	Yes	Yes	Yes	Yes	Yes	Yes
Observations	979	979	979	979	979	979	889	889
R2	0.097	0.097	0.099	0.099	0.063	0.064	0.289	0.289
Adjusted R2	0.054	0.054	0.057	0.058	0.021	0.021	0.256	0.256
Residual std. error	577.950	577.951	240.588	240.581	4.352	4.351	7.550	7.550
F statistic	2.271***	2.270***	2.386***	2.388***	1.511**	1.511**	8.624***	8.625***

Notes: The dependent variable is level of serum ferritin in µg/dL in regressions 1 and 2, serum retinol in µg/dL in regressions 3 and 4, inflammation status (CRP > 1 mg/dL) in regressions 5 and 6, and an indicator equal to 1 if the respondent's household has access to safe water in regressions 7 and 8 (0 otherwise). All models are estimated using sampling survey weights, and all specifications use a flood variable adjusted for district population density. Robust standard errors (in parenthesis) are clustered at the district level. * $p < 0.1$, ** $p < 0.05$, *** $p < 0.01$.
